# Personality Traits and Further Training

**DOI:** 10.3389/fpsyg.2020.510537

**Published:** 2020-11-16

**Authors:** Marie-Christine Laible, Silke Anger, Martina Baumann

**Affiliations:** ^1^Research Department Education, Training, and Employment Over the Life Course, Institute for Employment Research (IAB), Nuremberg, Germany; ^2^Chair of Economics, Economics of Education, Faculty of Social Sciences, Economics, and Business Administration, Otto-Friedrich University Bamberg, Bamberg, Germany; ^3^IZA Institute of Labor Economics, Bonn, Germany; ^4^Research Data Centre, Institute for Employment Research (IAB), Nuremberg, Germany

**Keywords:** socio-emotional skills, further training participation rates, NEPS, lifelong learning, continuing education, Big Five—personality

## Abstract

The notion of lifelong learning is gaining importance, not only in the labor market but also in other areas of modern societies. Previous research finds variation in occupation-related training participation by worker and workplace characteristics, gender, and education. However, evidence on the individual's socio-emotional skills creating favorable conditions for overall further training is scarce. To close this research gap, we analyze the role of personality for further training participation. First, we compare how the Big Five Personality Dimensions relate to different training types by differentiating between non-formal and informal training measures. Second, we investigate how personality traits affect further training chosen for occupational *and* private reasons separately. Drawing on a sample of 10,559 individuals from the Adult Stage of the German National Educational Panel Study (NEPS), we find that throughout our estimations, openness to experience positively relates to further training participation and is the most important determinant among the Big Five Personality Dimensions. However, the relationship between personality traits and training participation varies according to the training type and the reason for participating in further training. Moreover, we find gender-specific differences in the association between personality traits and lifelong learning. We conclude that personality is an important predictor of lifelong learning decisions.

## Introduction

Lifelong learning is continuously gaining importance, not only in the labor market, but also in private areas of modern societies. In the labor market, technological change, and additional dynamics through globalization and cyclical fluctuations lead to rapidly evolving work environments that require individuals to develop skills throughout their occupational careers (Acemoglu and Autor, 2011). At the same time, lifelong learning increases in importance, as both the shortage of skilled labor^1^ and demographic change require an increasingly later retirement age^2^, which—together with personal preferences of older persons to stay active—prolongs employment careers for older individuals (Anger et al., 2018).

Likewise, new technologies, and in particular digitalization, affect many areas outside of the labor market and entail a significant societal change with the requirement to continuously learn new techniques. Furthermore, these developments are accompanied by trends toward increasing individualization in modern societies. Individuals, especially in societies with a steadily growing life expectancy, depend on lifelong learning as a condition for social participation.

Hence, continuous investments in human capital through further training is a prerequisite to remain active in a modern society and productive in the labor market. The OECD promotes that “workers need a broad mix of skills—strong cognitive and socio-emotional skills, as well as digital skills” to successfully navigate the future of work (OECD, 2019, p. 3). These skills can only be developed, when “individuals acquire a good level of skills proficiency in initial education so they can develop these skills further over their lifetime as well as learn new skills along the way” (OECD, 2019, p. 40). Since initial skills—as condition for lifelong learning—may not be limited to cognitive abilities, the aim of this paper is to investigate the importance of non-cognitive skills for participation in further training.

### Socio-Emotional Skills, Personality Traits, and Their Development

A substantial body of literature considers socio-emotional skills and their influence on life outcomes. Socio-emotional skills “cover a wide range of personal characteristics such as personality traits, motivation, preferences and values” (Lechner et al., 2019a, p. 427). These characteristics have in common that they “can be (a) manifested in consistent patterns of thoughts, feelings and behaviors, (b) developed through formal and informal learning experiences, and (c) important drivers of socioeconomic outcomes throughout the individual's life” (OECD, 2015, p. 35).

Personality traits can be considered as a subset of socio-emotional skills (Kankaraš and Suarez-Alvarez, 2019, p. 9). They are defined as “relatively enduring, automatic patterns of thoughts, feelings, and behaviors that people exhibit in similar situations across time” (Roberts and Davis, 2016, p. 319)^3^. Like other socio-emotional skills, personality traits are in part developed by socializing and learning, and they have beneficial effects on individual education, work, and life success [for an overview, see e.g., Almlund et al. (2011), Brunello and Schlotter (2011)] as well as on societal outcomes (OECD, 2019). Personality traits can be conceived of as skills because they complement knowledge and transform cognitive skills into output (Cunningham et al., 2016, p. 7).

Many studies analyzing socio-emotional skills as determinants of life outcomes rely on the crucial assumption of stability in personality traits in adults to mitigate reverse causality concerns. In fact, however, ample evidence exists both for the malleability and for the stability of personality traits. Several studies have investigated whether personality traits change, to which extent they change, and how changes occur across the life course and in relation to specific life events [e.g., Roberts and DelVecchio (2000); Roberts et al. (2006); Specht et al. (2011); Damian et al. (2019)].

On the one hand, the literature concludes that genetics (partially) shape personality traits (Jang et al., 1996; Bouchard and Loehlin, 2001; Kandler et al., 2010) that develop throughout childhood and reach maturity in adulthood. Personality traits are shown to be increasingly stable over the life course until late middle age, when personality stability reaches a plateau (Roberts and DelVecchio, 2000; Soto, 2018), which can be mostly explained by a more stable environment (Briley and Tucker-Drob, 2014). On the other hand, previous studies find heterogeneous trait changes in childhood and adolescence, and substantial changes in young adulthood, with room for variability later in life (Roberts and Davis, 2016).

Summarizing the literature in its broad range, even if personality is not completely stable in adulthood and changes can take place throughout life (Roberts et al., 2006), the time-invariant component appears to outweigh the state-dependent component caused by situational fluctuations (Ferguson, 2010). Damian et al. (2019) confirm this finding in their study on the stability of personality traits over a 50-years-time span from adolescence to retirement age. While finding malleable personality traits across the whole life span, they acknowledge the stable component of personality. Over a much shorter time span, Specht et al. (2011) observe age effects on the Big Five Personality Dimensions for a large and representative longitudinal German sample, similar to ours, and show that changes in reaction to experiencing major life events occur in particular in young and old ages. Cobb-Clark and Schurer (2012) confirm largely stable Big Five personality traits in adulthood, particularly for working-age individuals. The literature largely agrees that few changes occur in older individuals (Costa et al., 2000; Srivastava et al., 2003), and even life-altering events such as unemployment are not observed to entail major changes in personality traits (Cobb-Clark and Schurer, 2012; Anger et al., 2017).

The literature also stresses gender differences in average traits (Bertrand, 2011). For example, Croson and Gneezy (2009) highlight differences in preferences and personality traits between men and women. Across nations, women score higher in agreeableness and conscientiousness and particularly in neuroticism (Costa et al., 2001).

### Socio-Emotional Skills and Life Outcomes

In the context of life outcomes, socio-emotional skills are treated as a part of an individual's human capital (Becker, 1964), which yields returns over the life cycle. In addition, in a behavioral model of wage setting, socio-emotional skills influence wage determination by shaping an individual's utility function (Bowles et al., 2001a,b). Moreover, Roberts et al. (2007) offer a theory explaining the association between personality traits and occupational success, which includes potential channels through which personality traits may affect occupational attainment. They distinguish between personality effects through niche finding, recruitment, environmental shaping, attrition, and direct performance (Roberts et al., 2007).

Lechner et al. (2019a) present a recent overview of the empirical relevance of socio-emotional skills for education and life outcomes^4^. Comparing the effects of personality and cognitive skills, Rammstedt et al. (2017) show a strong relationship between the Big Five personality measures and literacy and numeracy skills, implying that both skills “co-shape” life outcomes. In particular, conscientiousness and emotional stability contribute to explaining a wide range of economic and life outcomes—health, life satisfaction, educational attainment, continuing education, labor force participation, and income—beyond literacy and numeracy competencies. The contribution of personality varies with the life outcome: Personality explains a greater variation in life satisfaction and health than cognitive competencies. In contrast, the contribution of personality is lower for the economic outcomes income and employment status, as well as for education and continuing education compared to competencies.

Nevertheless, personality significantly contributes to explaining variation in continuing education. In a recent study, Lechner et al. (2019b) focus on the association of grit and career success and find that grit also positively relates to the amount of training taken.

One body of the literature focuses on the effect of socio-emotional skills on educational attainment. In particular, socio-emotional skills relate to educational achievement, such as grades and achievement tests (Poropat, 2009; Borghans et al., 2016; Vedel and Poropat, 2017). In addition, previous studies provide evidence on the effect of socio-emotional skills on educational transitions (Ng-Knight and Schon, 2017) and school dropout (Heckman et al., 2001; Coneus et al., 2011). Lundberg (2013b) examines the relationship between personality traits and high school graduation, college enrollment and college graduation. She finds that the returns to the Big Five personality traits vary by family background and that openness to experience, as the most important skill in this context, can substitute for having a less-advantaged parental background. Further, openness to experience also predicts successful college completion in the US, particularly for less-advantaged students, while conscientiousness has no significant effect (Lundberg, 2013a). Similar evidence exists for Germany, where the school to college transition is facilitated by openness to experience and emotional stability, and the intent to study in college is associated with both these traits (Peter and Storck, 2015). Additional evidence reveals that not only the school to college transition, but also the subject choice depends on personality traits (Berkes and Peter, 2019).

Focusing on labor market outcomes, Heckman et al. (2006) and Borghans et al. (2008) highlight the importance of non-cognitive skills in addition to cognitive skills for the determination of employment, work experience and occupational choice. There is vast evidence that personality does not only affect career choice, but also career development and attainment over the whole working life. More specifically, the Big Five personality traits are related to occupational attainment (Hogan and Holland, 2003), and evidence exists for long-term effects of extraversion, neuroticism, conscientiousness, and agreeableness on occupational status (Judge et al., 1999). In their meta-analysis of the determinants of career success, Roberts et al. (2007) show that the Big Five personality traits are strongly related to occupational attainment. More recent studies by Spengler et al. (2015) and Spengler et al. (2018) confirm these results and show that personality traits and student behaviors have direct and indirect effects on career success defined as occupational success and income.

Likewise, empirical studies on the relationship between personality traits and income demonstrate the importance of traits, such as for example leadership skills (Kuhn and Weinberger, 2005) and the Big Five personality traits, in particular extraversion (Sutin et al., 2009) and conscientiousness (Roberts et al., 2011). Even if measured early in life, personality traits are observed to impact earnings over the whole life span (Viinikainen et al., 2010). Thus, agreeableness for example is a favorable labor market trait, associated with better job performance (Barrick and Mount, 1991) and increasing the odds for re-employment after unemployment periods (Gnambs, 2017). However, some studies also find agreeableness to be punished through lower wages (Rode et al., 2008; Heineck, 2011; Judge et al., 2012).

Recent work emphasizes that employers value socio-emotional skills more than cognitive skills. It seems that employment and wage growth are stronger for jobs with high levels of both math and social skills, showing that cognitive skills and social skills are complementary (Deming, 2017; Deming and Kahn, 2018). According to the theoretical explanation, social skills reduce coordination costs and allow workers to specialize and work together better (Deming, 2017). Moreover, workers with higher social skills are observed to sort into non-routine and social-skill intensive occupations (Deming, 2017). Finally, firms that require these two skills also perform better (Deming and Kahn, 2018).

### Further Training

A separate strand of research investigates the determinants of further training participation. Previous studies on lifelong learning focus on the determinants of occupation-related further training and show that initial education has a significant impact on participation in further training over the life course (Kramer and Tamm, 2018). These studies also show that occupational training participation varies widely by worker type and workplace characteristics (Gerlach and Jirjahn, 2001; Brunello and Gambarotto, 2007; Rzepka and Tamm, 2016; Heß et al., 2019), by social group (Bilger, 2006; Leber and Möller, 2008), and by gender (Janssen and Wölfel, 2017), as well as with economic conditions (Bassanini and Brunello, 2008; Bellmann et al., 2014). When it comes to the choice of job-related training, time and financial constraints are crucial factors to deter individuals from training activities (Osiander and Stephan, 2018). While these studies focus on participation in occupational further training measures, scarce evidence exists on the determinants of general and non-work-related training activities.

Moreover, we know little about additional constraints for further training. An important constraint could be the lack of relevant non-cognitive skills, as insufficient socio-emotional skills may deter individuals from training participation. The scarce evidence on the importance of socio-emotional skills for further training activities focuses exclusively on occupational training: Caliendo et al. (2020) develop a theoretical model by including locus of control into the occupation-related training investment decisions. Using data from the German Socio-Economic Panel (SOEP) they reveal that locus of control relates to training participation through employee's expectations about future wage returns. The study closest to ours regards the Big Five personality traits and locus of control based on data from the SOEP (Offerhaus, 2012). In this study, agreeableness, extraversion and neuroticism do not affect occupation-related further training participation. In contrast, individuals who are open to new experiences and have a high internal locus of control are more likely to participate in work-related further training. However, existing studies do not differentiate between different types of training, for example course-based training vs. informal learning, which may be relevant, when it comes to personality traits as potential determinants of the initiation and continuity of different training activities. Furthermore, the importance of lifelong learning for social participation until an older age requires analyzing continuing education beyond occupation-related training.

### The Present Study

In summary, we know little about how non-cognitive skills affect further training decisions. This gap is in stark contrast to the substantial prior research on socio-emotional skills and their importance for predicting educational achievement, labor market success and a broad range of life outcomes (Heckman et al., 2006; Borghans et al., 2008; Almlund et al., 2011; Heckman and Kautz, 2012; Lechner et al., 2019a). Previous studies point to increasing returns to socio-emotional skills over the past decades, specifically as complements to cognitive skills (Brunello and Schlotter, 2011; Deming, 2017; Edin et al., 2017). This increase may at least partially be driven by the growing importance of further training participation, which may be affected by socio-emotional skills.

Likewise, we know little about the effects of personality traits as a subdomain of socio-emotional skills on lifelong learning. Exceptions are the two aforementioned studies focusing on employment-related training activities without further specification of the training type. The participation in occupational further training is affected by both locus of control (Caliendo et al., 2020) and openness to experience (Offerhaus, 2012). However, given the need for continuous investments in human capital to adapt to changing environments both inside and outside of the labor market, it is important to understand which socio-emotional skills act as barriers or promote lifelong learning in general.

To close this research gap, we provide an in-depth analysis of the role of personality traits for further training participation. We focus on the Big Five Personality Dimensions and investigate first whether the relationship between personality and further training varies by training type. Differentiating between non-formal training (i.e., course-based training without a formal degree) and informal training (i.e., training without structured coursework), may be relevant, as the different training types differ in their requirement for training initiation, involvement, intensity, and continuity. As a result, personality traits may have a different impact on training for different training types. Likewise, the differentiation between employment-related training and lifelong learning for private reasons is important, as personality traits may matter differently for the participation of training inside and outside of the work environment.

Using the Adult Stage of the National Educational Panel Study (NEPS), we show that the Big Five Personality Dimensions significantly relate to further training activities, both for overall further training participation and for specific training types (i.e., differentiating between non-formal and informal training). For non-formal training, we separately look at the reasons to partake in a training activity (i.e., private as opposed to occupationally motivated reasons). The overall pattern of our results indicates that no matter which type of, or reasons for, training we analyze, openness to experience positively relates to further training participation and is the most important determinant of training activities. When differentiating between training types and when estimating separate regressions by gender, different patterns for the Big Five emerge.

Our study adds to the scarce literature on personality traits as determinants of further training participation. In addition to validating prior results on the importance of openness to experiences for occupation-related further training (Offerhaus, 2012), we expand the existing research in several ways. First, we take advantage of the high-quality data provided by the NEPS Adult Cohort study. By using this panel survey, we make use of the yearly measurements of the same individuals, both by averaging repeated measurements to reduce bias from measurement error and by accounting for unobservable heterogeneity when applying panel estimators. We exploit the detailed NEPS questions on different types of further training, as well as its distinction between different reasons for investing in continuous training. Thereby, we analyze whether different personality traits are relevant for non-formal and informal training, as well as for private compared to work-related further training decisions. Second, we use recent survey data, allowing the estimation of the relationship between personality traits and further training in current labor market conditions and societal dynamics, which are shaped by digitalization, demographic changes and a post-recession period. These rapid changes may affect the association between personality traits and training participation over time, possibly revealing that patterns observed in prior studies are changing. Third, we account for average personality differences between men and women and allow for potential gender differences in the association between personality type and training activity.

## Methods

### The Data

We use longitudinal data from the German National Educational Panel Study (NEPS), which collects information on complete educational biographies, transitions in educational careers, and lifelong learning on an annual basis since 2008. The NEPS surveys individuals in six starting cohorts from newborn infants to adults (Anger et al., 2019), and uses short recall periods to the previous interview and assists respondents in remembering their activities through recall help. For example, preloads are integrated into the questionnaire of the current interview to help respondents anchor their answers^5^. These procedures make the data very reliable and ensure that information is correctly measured.

To investigate the effect of personality traits on further training, we use the scientific use file NEPS SUF SC 9.0.1 for the Adult Stage (Starting Cohort 6—SC6, Stage 8)^6^. The Adult Cohort is based on the population of working-age adults (in or out of employment) in Germany, born between 1944 and 1986. The respondents are asked about their life course with a focus on lifelong learning and further training.

### The Participants

We restrict our sample to wave 5 (Fall 2012 to Spring 2013) and wave 8 (Fall 2015 to Spring 2016) because the Big Five are only surveyed in those years. We further exclude respondents below the age of 25 and above the age of 65 to ensure that the individuals have mostly finished their initial education and are potentially susceptible for further training. Finally, we only include individuals for whom non-missing information on further training participation or non-participation is available for both non-formal and informal training activities^7^. In our full estimation sample with all training types and reasons for training, we thus include 17,242 individual-year observations from 10,559 individuals, of which 6,683 provide the relevant information in both waves.

Table 1 reports the summary statistics for the pooled sample. They show that a little more than half of the sample is female (N_female_ = 5,325; N_male_ = 5,234) and the average age in wave 8 is around 49,6 years. The respondents in the sample are relatively highly educated: A high share of all respondents have an intermediate secondary degree (33%— “Realschule”) or a high school degree (48%— “Abitur”). Men more often have a high school degree than women, whereas among women, an intermediate secondary degree is more widespread than among men.

**Table 1:** Summary statistics.

	**Full Sample**			**Males**			**Females**		
	**Mean**	***SD***	**95% CI**	**Mean**	***SD***	**95% CI**	**Mean**	***SD***	**95% CI**
Further training (overall)	0.77	0.42	[0.77, 0.78]	0.79	0.40	[0.79, 0.80]	0.76	0.43	[0.75, 0.77]
Non-formal training	0.40	0.49	[0.40, 0.41]	0.38	0.48	[0.37, 0.39]	0.43	0.50	[0.42, 0.44]
- Privately motivated	0.27	0.45	[0.26, 0.28]	0.23	0.42	[0.22, 0.25]	0.31	0.46	[0.30, 0.33]
Informal training	0.69	0.46	[0.68, 0.69]	0.72	0.45	[0.71, 0.73]	0.66	0.47	[0.65, 0.67]
Age	49.56	9.60	[49.34,49.77]	49.39	9.44	[49.09, 49.67]	49.73	9.77	[49.42, 50.05]
Gender				0.49			0.51		
Education									
- No degree	<0.01			<0.01			<0.01		
- Lower secondary degree	0.18			0.20			0.16		
- Intermediate secondary degree	0.33			0.28			0.38		
- High school degree	0.48			0.51			0.46		
*N*	17,242			8,532			8,710		

*Unweighted. Pooled data. Means and standard deviations (SD); 95% confidence intervals (CI). Gender and age in Wave 8. Rounded percentages for education. Source: Own calculations based on NEPS SUF SC6 9.0.1*.

### The Measures

#### Further Training Types

We include information on different further training measures that the survey annually asks about. We follow the definition of Eisermann et al. (2014) and distinguish between three types of further training: First, formal further training includes all training activities after initial education, which lead to a formal degree. Initial education can be defined in different ways but usually refers to the educational career until the first employment spell or until an interruption of schooling of more than 12 months (Kruppe and Trepesch, 2017). We refrain from estimating specifications with formal further training as dependent variable because very few adults participate in this training form each year. Second, non-formal training comprises all organized training activities, which may or may not lead to a certificate. Third, informal training is defined as non-structured further training, such as on-the-job training, reading professional literature, visiting conferences, or lectures and using self-learning programs.

Table 2 provides an overview of the different training types, their definitions, sample questions from the questionnaire, and examples of what a specific type of training might be.

**Table 2:** Definition and examples for training types and motives.

	**Definition**	**Item in questionnaire (non-comprehensive)**	**Example of training**
**Training type**				
Formal	Any kind of further training after initial education, which may be a continuation or reuptake of learning activities that lead to a generally accepted degree or to a certified qualification	Now let's talk about your school education. Have you attended a general educational school since <last interview date>. (Please also consider general educational schools of the second chance education type, such as evening schools.)	High school degree, master tradesman's or craftsman's certificate, bachelor or master degree
Non-formal	Specifically organized, course-based training or seminars with or without certificates and without a generally accepted degree	Let's return to the subject of further training. Up until now you have stated that, since the last interview, you attended the following courses or training programs: <list of courses> Since the last interview, have you, in addition to this, i.e., from <last interview date> to the present, attended courses or training programs that you have not yet mentioned?	IT (Excel, Word, etc.), project management, law, cooking, yoga, languages
Informal	Non-organized learning activities that do not lead to a certification or degree; often self-organized training, on-the-job-training	Learning may also be done completely without regulated class and course routines. Since the last interview in <last interview date> did you visit special trade fairs or congresses, to learn more on your own in the professional or private field?	Trade fairs, conferences, professional talks or lectures, professional literature (books and journals), learning CDs or DVDs
**Training motive (only available for non-formal training)**			
Private	Non-formal training taken for private purposes only	Did you attend this course primarily for professional reasons or rather out of personal reasons? YES	Cooking, yoga, languages
Work-related	Non-formal training taken for employment-related purposes	Did you attend this course primarily for professional reasons or rather out of personal reasons? NO	IT (Excel, Word, etc.), project management, law

*Text between < and > refers to Preloads, i.e., the date of the previous interview or a list of previously mentioned training. Definition according to Eisermann et al. (2014). Sample items from questionnaire from https://www.neps-data.de/Portals/0/NEPS/Datenzentrum/Forschungsdaten/SC6/10-0-0/SC6_10-0-0_W10_en.pdf. Initial training is defined as any education spell up to the first employment spell or an educational spell up to a break of more than 12 months (Kruppe and Trepesch, 2017)*.

In addition to this threefold definition, the NEPS provides information on the motivation for participating in a non-formal training activity (i.e., whether the training was privately or occupationally motivated)^8^. As these additional questions are only asked for a random sample of non-formal training activities, the number of observations decreases for this sample to 5,067 individuals.

The summary statistics for the pooled sample in Table 1 shows that in the full estimation sample, around 77% of all respondents participated in further training of any type in wave 5 or 8. Men have a slightly higher participation rate compared to women (79 vs. 76%)^9^. Approximately 40% of the respondents attend non-formal training, while 69% pursue informal training. Fewer respondents (27%) participate in privately motivated further training.

#### Personality Traits

The personality traits we analyze are the Big Five Personality Dimensions. This psychological concept categorizes an individual's personality into five traits: Extraversion, neuroticism, agreeableness, conscientiousness and openness to experience. Each trait consists of characteristics that describe the personality dimension. The personality traits are measured by the well-established “Big Five Inventory Short Scale,” the BFI-10 (Rammstedt and John, 2007). This scale includes 11 items asking the respondent to answer on a five-point Likert scale ranging from “*fully disagree*” to “*fully agree*.” Each trait is measured by two items with the exception of agreeableness, which is measured by three items (Table 3, Column 2).

**Table 3:** The Big Five Personality Dimensions and associated traits.

**Big five dimension**	**Item**	**Cronbach's alpha** **and Revelle's omega**
Introversion vs. extraversion	is reserved is outgoing, sociable	Alpha: 0.66 Omega: 0.66
Antagonism vs. agreeableness	tends to find fault with others is generally trusting is considerate and kind to almost everyone	Alpha: 0.35 Omega: 0.41
Lack of direction vs. conscientiousness	tends to be lazy does a thorough job	Alpha: 0.43 Omega:0.43
Emotional stability vs. neuroticism	is relaxed, handles stress well gets nervous easily	Alpha: 0.49 Omega: 0.49
Closed to experience vs. openness to experience	has few artistic interests has an active imagination	Alpha: 0.47 Omega: 0.47

*Source: NEPS Adult Stage Questionnaire following BFI-10 (Rammstedt and John, 2007). Own calculations based on NEPS SUF SC6 9.0.1 using the R psych package. Number of observations is 17,242*.

To evaluate internal consistency, we compute Cronbach's Alpha and Revelle's Omega for each of the Big Five Personality Dimensions provided by the NEPS (Table 3, Column 3). Since the Cronbach's Alphas are “a function of the mean inter-item correlation and the number of items comprising the scale” (Gosling et al., 2003, p. 516) and given that our Big Five measures consist of only two or three items per trait, it is not surprising that the Alphas are only of moderate size. The Omegas confirm the results obtained through Cronbach's Alpha. Nevertheless, we follow Rammstedt and John (2007) and Gosling et al. (2003) in their assessments that short Big Five scales are valid, reliable and good proxies for longer scales.

Table 4 displays the means and standard deviations of the Big Five traits for the two available waves for individuals with non-missing information on personality in both waves^10^. The virtually identical mean levels of the Big Five Personality Dimensions show that the personality traits on average do not vary much for the whole sample within the 3-years' time interval.

**Table 4:** Means, standard deviations and intra-individual correlations of the Big Five Personality Dimensions.

	**Wave 5**			**Wave 8**			**Intra-individual correlation across waves**	
	**Mean**	***SD***	**95% CI**	**Mean**	***SD***	**95% CI**	**CC (*p*-values)**	**95% CI**
Extraversion	3.376	0.919	[3.354, 3.398]	3.381	0.881	[3.359, 3.402]	0.653*** (<0.001)	[0.639; 0.667]
Agreeableness	3.577	0.589	[3.562, 3.591]	3.565	0.565	[3.551, 3.578]	0.542*** (<0.001)	[0.525; 0.559]
Conscientiousness	4.028	0.714	[4.011, 4.045]	3.981	0.687	[3.965, 3.998]	0.581*** (<0.001)	[0.565; 0.596]
Neuroticism	2.573	0.798	[2.554, 2.593]	2.620	0.776	[2.602, 2.639]	0.544*** (<0.001)	[0.526; 0.560]
Openness	3.480	0.908	[3.458, 3.502]	3.403	0.897	[3.381, 3.424]	0.625*** (<0.001)	[0.611; 0.640]
*N*	6,683			6,683			6,683	

*Means and standard deviations (SD). Correlation coefficient (CC) only for individuals with non-missing observations in both waves. Non-standardized personality traits. Unweighted. Pooled data*.

*p < 0.05, **p < 0.01, ***p < 0.001.

*Source: Own calculations based on NEPS SUF SC6 0.0.1*.

However, mean-level changes for the whole sample may disguise individual variation in personality traits over time due to offsetting changes in a particular trait dimension among individuals [e.g., Roberts (1997), Roberts and DelVecchio (2000)], since personality may vary with specific events or with increasing age [e.g., Roberts and DelVecchio (2000), Roberts et al. (2006), Specht et al. (2011), Damian et al. (2019)], in particular given the relatively large age-range in our sample. Hence, we additionally consider intra-individual changes in personality traits across the two survey waves, and report correlations between wave 5 and wave 8 in the last column of Table 4. The intra-individual correlations of openness to experience and extraversion are fairly high (>0.6), while the correlations of the other personality traits are moderate (around 0.55). Given the relatively short time span of only 3 years, we attribute the observed fluctuations mainly to the measurement error from calculating the personality traits based on the two or maximum three items provided by the NEPS. Taken together with the finding in the literature that personality stability reaches a plateau in late middle age (Roberts and DelVecchio, 2000; Soto, 2018), we conclude that the personality traits, and in particular openness to experience and extraversion, do not drastically change in our sample.

Thus, we focus on the core of personality and calculate the averages of the Big Five personality measures across the two waves for individuals with two observations in our sample to use these calculated means for all waves. This allows us to proxy for the part of personality that is relatively stable over time by netting out the time-variant component caused by situational fluctuations and to reduce possible measurement error (Zimmerman, 1992). Since we acknowledge that variability in the traits is truly possible and cannot rule out significant changes in personality traits in our sample, we additionally use the wave-specific measures of the Big Five Personality Dimensions and hence also estimate the effects of time-varying personality traits on further training in our multivariate estimations.

Finally, we recognize that personality may differ between individuals at different stages in the human lifecycle and therefore use age-corrected personality measures^11^. We follow the method by Nyhus and Pons (2012) and regress each trait on age and age squared to use the predicted residuals as “age-free” measures for the analyses. This procedure picks up possible maturity and feedback effects on personality over the lifecycle, for example via an individual's job and the social environment. We normalize each Big Five trait to a mean of zero and a standard deviation of one for each wave and generate an index, which is better able to reflect the continuum of personality and allows an easier interpretation of the results.

Table 5 compares the standardized age-corrected Big Five personality measures of further training participants and non-participants used in our multivariate analyses. The *t*-tests to examine whether participants of further training activities and non-participants significantly differ in their average personality traits reveal that participants and non-participants significantly differ in four dimensions. At this descriptive level, training participants are on average more extroverted and indicate a higher level of openness to new experience, while they appear to be less conscientious and less neurotic than non-participants.

**Table 5:** Standardized, age-corrected Big Five Personality Dimensions of further training participants and of non-participants.

	**(1)**		**(2)**		**(3)**	
	**With further training participation**		**Without further training participation**		***t*****-test**	
	**Mean**	**95% CI**	**Mean**	**95% CI**	***t*-value (*p*-value)**	**95% CI**
Extraversion	0.029	[0.012; 0.046]	−0.099	[−0.132; −0.067]	−7.05*** (<0.001)	[−0.164, −0.093]
Agreeableness	0.007	[−0.009; 0.024]	−0.025	[−0.059; 0.008]	−1.77 (0.076)	[−0.068, 0.003]
Conscientiousness	−0.009	[0.026; 0.008]	0.031	[−0.002; 0.063]	2.18** (0.029)	[0.004, 0.075]
Neuroticism	−0.026	[−0.043; −0.010]	0.090	[0.056; 0.124]	6.40*** (<0.001)	[0.081, 0.152]
Openness	0.089	[0.072; 0.105]	−0.305	[−0.337; −0.274]	−21.92*** (<0.001)	[−0.429, −0.359]
*N*	13,361		3,881		17,242	

*Standardized age-corrected personality traits. Unweighted. Pooled data. Individuals may fall into different categorie (with/without further training participation) across waves*.

*p < 0.5, **p < 0.01, ***p < 0.001.

*Source: Own calculations based on NEPS SUF SC6 9.0.1*.

#### Control Variables

We use a set of covariates to reduce potential biases from confounding variables or selection when estimating the relationship between the Big Five and further training. Thus, we control for demographic variables, namely gender, age and education (no degree, lower secondary degree, intermediate secondary degree, high school degree), as they relate to the Big Five and further training participation. Furthermore, we control for the presence of children under 6 years living in the household, household income and unemployment, as these characteristics may affect the respondents in their ability to participate in further training. We additionally control for the survey wave.

### Statistical Analysis

#### The Binary Outcome Model

We estimate binary outcome models, where an individual *i* either takes part in a training activity in a particular wave *t* or not:

$$\begin{array}{l} {FTP}_{it}=\left\{ \begin{array}{l} 1\;if\;i\;participates\;in\;further\;training\;in\;wave\;t\\ 0\;if\;i\;does\;not\;participate\;in\;further\;training\;in\;wave\;t \end{array}\right. \end{array}$$

As we estimate the predicted probabilities of different training outcomes, *FTP*_*it*_ is a dummy for either (1) overall further training participation, (2) non-formal further training participation, or (3) informal further training participation. For non-formal further training, we additionally differentiate in (4) privately motivated further training participation as opposed to occupation-related training.

Our underlying assumption is that individuals choose to invest in further training, if their expected returns from participating in this training are higher than their costs. The costs can be monetary or non-monetary, such as time and effort expanded in the training. In addition to standard determinants of educational investments, such as age, personality traits may influence this cost-benefit calculation. We focus on the effect of the Big Five Personality Dimensions in our analyses and estimate a binary choice model of the following form:

$$\begin{array}{l} {FTP}_{it}=a_{0}+{\text{B}\text{F}}_{i}a_{1}+{x^{\prime }}_{it}a_{2}+\varepsilon_{it} \end{array}$$

where *FTP*_*it*_ is the further training participation dummy for the different training types chosen by individual *i* in survey wave *t*. It equals 1 if the individual participates in further training, and zero otherwise. Because we assume in a first specification that the Big Five Personality Dimensions are stable in adults and use the mean personality trait across the available waves, the Big Five Personality Dimensions *BF*_*i*_ are time-invariant in most of our analyses^12^. The vector *X* controls for gender and for the time-varying individual characteristics of age, education, the presence of children under 6 years of age in the household, unemployment and household income. We also include time dummies in the estimations to control for wave-specific differences. The error term ε_*it*_ is clustered at the individual level.

#### The Estimation Techniques

To gain a preliminary understanding of the importance of personality traits for further training, we start by estimating linear probability models. In a first step, we do not leverage the time variation in further training and use a pooled ordinary least squares estimator (OLS) where we use all waves of each individual without accounting for the different waves. This estimator calculates marginal effects directly and is used for ease of interpretation. In a second step, we estimate Random Effects (RE) OLS models to exploit the time variation in further training and account for unobserved heterogeneity. Any variables that are not observed in the data (i.e., unobserved heterogeneity), may be problematic if they correlate with our variables of interest. Unobserved variables potentially cause omitted variable bias, meaning our results are attributed to personality when they should be attributed to the omitted variable. Potential omitted variables in our sample might be motivation or ability. However, by using panel estimation techniques, we are able to control for these unobserved factors through an individual-specific error term capturing all unobserved time-invariant heterogeneity and thereby producing consistent results^13^.

While OLS estimators are preferable because of their ease of interpretation, their caveat is that they rely on the dependent variable being continuous. However, our dependent variables only have two outcomes, namely the participation in a further training or the non-participation. Therefore, we refine the models by using a non-linear specification and choose an estimator with a normal distribution assumption, the Probit estimator. This estimator's coefficients do not directly yield marginal effects. As we are interested in the ceteris paribus effect that a change in a personality trait has on the predicted probability of further training participation, we calculate average marginal effects and present these in the tables.

The Pooled Probit estimator has the advantage that we may compare our results with these from the prior literature. However, as these results may be biased due to unobserved factors (i.e., omitted variable bias), we prefer specifications which exploit the panel data. Therefore, we take advantage of the additional information in the time variation and control for unobserved heterogeneity by estimating Random Effects Probit models.

As mentioned before, we follow the 2-fold strategy of first using the means of the Big Five measures over time to capture the stable part of personality and to reduce the potential bias resulting from measurement error, and second, estimating regressions based on time-varying Big Five measures to allow for variability in personality.

## Results

### Overall Training Participation

First, we analyze the effect of the Big Five Personality Dimensions on overall training participation. This measure includes all non-formal and informal as well as work-related and private training activities. Table 6 presents the coefficients for the pooled OLS and the RE estimations (panel A). We then show the average marginal effects for the Pooled Probit and RE Probit estimations (panel B). For each model, we first show the results without control variables and the results with controls in the adjoining column. We present results from OLS regressions for comparison with previous studies and as these allow for a more intuitive interpretation, but prefer the Probit specification, as this models the data more correctly. For either method, the resulting marginal effects are quite similar and therefore we merely focus on the Probit results in the following tables.

**Table 6:** Big Five Personality Dimensions and participation in overall further training.

**Panel A: Pooled OLS (top) and Random Effects (bottom)**						
	**Pooled OLS model**					
	**Model 1**			**Model 2**		
	**Average ME**	***p*****-value**	**95% CI**	**Average ME**	***p*****-value**	**95% CI**
Extraversion	0.0123***	(<0.001)	[0.00572, 0.0190]	0.0139***	(<0.001)	[0.00757, 0.0203]
Agreeableness	0.00236	(0.476)	[−0.00413, 0.00886]	0.00488	(0.126)	[−0.00137, 0.0111]
Conscientiousness	−0.0133***	(<0.001)	[−0.0197, −0.00687]	−0.00380	(0.227)	[−0.00995, 0.00236]
Neuroticism	−0.0102**	(0.003)	[−0.0169, −0.00348]	−0.00349	(0.287)	[−0.00992, 0.00294]
Openness	0.0696***	(<0.001)	[0.0632, 0.0759]	0.0540***	(<0.001)	[0.0479, 0.0602]
Age	0.0217***	(<0.001)	[0.0165, 0.0270]	0.00871**	(0.001)	[0.00348, 0.0139]
Age^2^	−0.000277***	(<0.001)	[−0.000334, −0.000220]	−0.000123***	(<0.001)	[−0.000180, −0.0000666]
Gender	−0.0509***	(<0.001)	[−0.0639, −0.0379]	−0.0433***	(<0.001)	[−0.0558, −0.0308]
Additional controls	No			Yes		
*N*	17,242			17,242		
	**Random Effects model**					
Extraversion	0.0120***	(0.001)	[0.00507, 0.0190]	0.0137***	(<0.001)	[0.00708, 0.0204]
Agreeableness	0.00357	(0.302)	[−0.00321, 0.0104]	0.00552	(0.098)	[−0.00102, 0.0121]
Conscientiousness	−0.00989**	(0.004)	[−0.0165, −0.00325]	−0.00212	(0.515)	[−0.00850, 0.00426]
Neuroticism	−0.00941**	(0.008)	[−0.0163, −0.00249]	−0.00382	(0.260)	[−0.0105, 0.00283]
Openness	0.0622***	(<0.001)	[0.0554, 0.0690]	0.0492***	(<0.001)	[0.0427, 0.0557]
Age	0.0209***	(<0.001)	[0.0153, 0.0266]	0.00913**	(<0.001)	[0.00356, 0.0147]
Age^2^	−0.000269***	(<0.001)	[−0.000330, −0.000208]	−0.000127***	(<0.001)	[−0.000187, −0.0000668]
Gender	−0.0511***	(<0.001)	[−0.0656, −0.0366]	−0.0438***	(<0.001)	[−0.0576, −0.0301]
Additional controls	No			Yes		
*N*	17,242			17,242		
**Panel B: Pooled Probit (top) and Random Effects Probit (bottom)**						
	**Pooled OLS model**					
	**Model 1**			**Model 2**		
	**Average ME**	***p*****-value**	**95% CI**	**Average ME**	***p*****-value**	**95% CI**
Extraversion	0.0123***	(0.001)	[0.00535, 0.0192]	0.0132***	(<0.001)	[0.00675, 0.0197]
Agreeableness	0.00323	(0.352)	[−0.00358, 0.0100]	0.00539	(0.096)	[−0.000966, 0.0117]
Conscientiousness	−0.0134***	(<0.001)	[−0.0202, −0.00650]	−0.00361	(0.268)	[−0.00999, 0.00277]
Neuroticism	−0.0100**	(0.005)	[−0.0170, −0.00309]	−0.00312	(0.343)	[−0.00955, 0.00332]
Openness	0.0682***	(<0.001)	[0.0616, 0.0747]	0.0518***	(<0.001)	[0.0456, 0.0580]
Age	0.0199***	(<0.001)	[0.0143, 0.0254]	0.00801**	(0.005)	[0.00247, 0.0136]
Age^2^	−0.000255***	(<0.001)	[−0.000314, −0.000195]	−0.000112***	(<0.001)	[−0.000171, −0.0000532]
Gender	−0.0499***	(<0.001)	[−0.0643, −0.0355]	−0.0420***	(<0.001)	[−0.0556, −0.0285]
Additional controls	No			Yes		
*N*	17,242			17,242		
	**Random Effects model**					
Extraversion	0.0122***	(<0.001)	[0.00540, 0.0190]	0.0131***	(<0.001)	[0.00674, 0.0195]
Agreeableness	0.00431	(0.202)	[−0.00231, 0.0109]	0.00599	(0.059)	[−0.000225, 0.0122]
Conscientiousness	−0.0106**	(0.002)	[−0.0173, −0.00391]	−0.00246	(0.442)	[−0.00874, 0.00381]
Neuroticism	−0.00925**	(0.007)	[−0.0160, −0.00254]	−0.00339	(0.289)	[−0.00966, 0.00288]
	**Random Effects model**					
	**Model 1**			**Model 2**		
	**Average ME**	***p*****-value**	**95% CI**	**Average ME**	***p*****-value**	**95% CI**
Openness	0.0616***	(<0.001)	[0.0551, 0.0681]	0.0476***	(<0.001)	[0.0414, 0.0538]
Age	0.0197***	(<0.001)	[0.0141, 0.0252]	0.00857**	(0.002)	[0.00308, 0.0141]
Age^2^	−0.000252***	(<0.001)	[−0.000311, −0.000193]	−0.000117***	(<0.001)	[−0.000176, −0.0000592]
Gender	−0.0500***	(<0.001)	[−0.0644, −0.0356]	−0.0419***	(<0.001)	[−0.0554, −0.0284]
Additional controls	No			Yes		
*N*	17,242			17,242		

*Average marginal effects (ME) with p-values in parentheses and confidence interval (CI) in square brackets*.

*p < 0.05, ** p < 0.01, *** p < 0.001.

*Panel A: Average marginal effects of pooled ordinary least squares (OLS) and random effects estimation. Robust standard errors clustered at the individual level (10,559 individuals)*.

*Panel B: Average marginal effects of pooled probit and random effects probit estimation. Robust standard errors are clustered at the individual level (10,559 individuals)*.

*Model 1 in each panel contains the following control variables: Gender (female = 1), age, and a wave indicator. Model 2 contains the following additional control variables: Children under six years in the household (yes = 1), education (no degree, lower secondary degree, intermediate secondary degree, high school degree), household income, unemployment (yes = 1)*.

*Source: Own calculations based on NEPS SUF SC6 9.0.1*.

With respect to the control variables, Table 6 reveals that in all models and specifications, women are significantly less likely to participate in further training compared to men. We also find that the likelihood to participate in further training significantly relates to age. This relationship is curvilinear with a peak at about 44 years of age in the model with additional controls. For our main variables of interest, the estimates show that extraversion and openness to experience positively relate to the predicted probability to participate in further training even after the inclusion of additional control variables. In contrast, the remaining Big Five Personality Dimensions are not significantly associated with further training participation in the specification with controls.

Since we expect a bias in the pooled estimations due to unobserved factors that may affect the outcome, we exploit the panel character of the data and control for time-invariant unobserved individual heterogeneity in the Random Effects Probit estimations. The results confirm the pattern from the pooled estimations, such that extraversion and openness to new experiences positively relate to the dependent variable. In the RE Probit estimation, the effect size of openness to experience and extraversion is slightly smaller compared to the Probit model without Random Effects. Notably, the marginal effect for openness to experience is generally larger in magnitude compared to the other personality traits.

### Non-formal and Informal Further Training

We exploit the detailed information on further training available in the NEPS and differentiate in the next step between the different training types. Thus, we run separate estimations for non-formal and informal further training participation to assess whether personality traits equally relate to participation probabilities for organized training activities (non-formal further training) and self-organized and less structured further education (informal further training). Note that informal training is likely to drive the overall results of Table 6, as 69% of all respondents participate in informal further training, while only 40% participate in non-formal further training (as indicated by Table 1). Table 7 presents the results for non-formal further training participation and for informal further training participation. When differentiating between further training types, we decrease the information density in the dependent variable (1 = any training vs. 1 = only non-formal (informal) training) leading to less precise estimations.

**Table 7:** Big Five Personality Dimensions and non-formal and informal further training participation.

**Panel A: Participation in non-formal further training**						
	**Pooled OLS model**					
	**Model 1**			**Model 2**		
	**Average ME**	***p*****-value**	**95% CI**	**Average ME**	***p*****-value**	**95% CI**
Extraversion	0.0141^***^	(<0.001)	[0.00621, 0.0221]	0.0143^***^	(<0.001)	[0.00652, 0.0222]
Agreeableness	0.0109^**^	(0.005)	[0.00327, 0.0186]	0.0132^***^	(0.001)	[0.00559, 0.0208]
Conscientiousness	−0.0123^**^	(0.002)	[−0.0201, −0.00445]	−0.00710	(0.073)	[−0.0148, 0.000653]
Neuroticism	−0.0103^*^	(0.010)	[−0.0181, −0.00243]	−0.00547	(0.167)	[−0.0132, 0.00229]
Openness	0.0402^***^	(<0.001)	[0.0324, 0.0480]	0.0320^***^	(<0.001)	[0.0243, 0.0398]
Age	0.0318^***^	(<0.001)	[0.0253, 0.0383]	0.0205^***^	(<0.001)	[0.0139, 0.0271]
Age^2^	−0.000380^***^	(<0.001)	[−0.000449, −0.000310]	−0.000250^***^	(<0.001)	[−0.000321, −0.000179]
Gender	0.0449^***^	(<0.001)	[0.0285, 0.0614]	0.0521^***^	(<0.001)	[0.0359, 0.0683]
Additional controls	No			Yes		
*N*	17,242			17,242		
	**Random Effects model**					
Extraversion	0.0135^***^	(0.001)	[0.00565, 0.0213]	0.0139^***^	(<0.001)	[0.00620, 0.0217]
Agreeableness	0.0105^**^	(0.006)	[0.00297, 0.0181]	0.0127^***^	(0.001)	[0.00525, 0.0202]
Conscientiousness	−0.0112^**^	(0.004)	[−0.0189, −0.00350]	−0.00650	(0.096)	[−0.0142, 0.00116]
Neuroticism	−0.0110^**^	(0.005)	[−0.0187, −0.00329]	−0.00651	(0.096)	[−0.0142, 0.00116]
Openness	0.0393^***^	(<0.001)	[0.0316, 0.0469]	0.0314^***^	(<0.001)	[0.0237, 0.0391]
Age	0.0322^***^	(<0.001)	[0.0258, 0.0386]	0.0212^***^	(<0.001)	[0.0146, 0.0278]
Age^2^	−0.000385^***^	(<0.001)	[−0.000453, −0.000316]	−0.000259^***^	(<0.001)	[−0.000329, −0.000188]
Gender	0.0449^***^	(<0.001)	[0.0286, 0.0613]	0.0518^***^	(<0.001)	[0.0357, 0.0679]
Additional controls	No			Yes		
*N*	17,242			17,242		
**Panel B: Participation in informal further training**						
	**Pooled Probit model**					
	**Model 1**			**Model 2**		
	**Average ME**	***p*****–value**	**95% CI**	**Average ME**	**p–value**	**95% CI**
Extraversion	0.0101*	(0.011)	[0.00236, 0.0179]	0.0125^***^	(0.001)	[0.00524, 0.0199]
Agreeableness	0.00276	(0.475)	[−0.00482, 0.0103]	0.00505	(0.162)	[−0.00203, 0.0121]
Conscientiousness	−0.0110^**^	(0.005)	[−0.0186, −0.00338]	0.00114	(0.753)	[−0.00598, 0.00826]
Neuroticism	−0.0100^*^	(0.011)	[−0.0178, −0.00231]	−0.00250	(0.496)	[−0.00970, 0.00470]
Openness	0.0793^***^	(<0.001)	[0.0721, 0.0866]	0.0596^***^	(<0.001)	[0.0526, 0.0666]
Age	0.0145^***^	(<0.001)	[0.00808, 0.0208]	0.00323	(0.313)	[−0.00304, 0.00951]
Age^2^	−0.000190^***^	(<0.001)	[−0.000258, −0.000122]	−0.0000509	(0.135)	[−0.000118, 0.0000158]
Gender	−0.0742^***^	(<0.001)	[−0.0904, −0.0581]	−0.0664^***^	(<0.001)	[−0.0816, −0.0512]
Additional controls	No			Yes		
*N*	17,242			17,242		
	**Random Effects Probit model**					
Extraversion	0.0108^**^	(0.005)	[0.00323, 0.0183]	0.0128^***^	(<0.001)	[0.00572, 0.0200]
Agreeableness	0.00439	(0.240)	[−0.00293, 0.0117]	0.00610	(0.083)	[−0.000796, 0.0130]
Conscientiousness	−0.00831^*^	(0.026)	[−0.0157, −0.000973]	0.00178	(0.614)	[−0.00515, 0.00872]
Neuroticism	−0.00788^*^	(0.036)	[−0.0153, −0.000495]	−0.00196	(0.580)	[−0.00892, 0.00500]
	**Random Effects Probit Model**					
	**Model 1**			**Model 2**		
	**Average ME**	***p*****–value**	**95% CI**	**Average ME**	**p–value**	**95% CI**
Openness	0.0686^***^	(<0.001)	[0.0614, 0.0759]	0.0526^***^	(<0.001)	[0.0456, 0.0595]
Age	0.0137^***^	(<0.001)	[0.00737, 0.0199]	0.00344	(0.275)	[−0.00274, 0.00962]
Age^2^	−0.000182^***^	(<0.001)	[−0.000249, −0.000115]	−0.0000526	(0.116)	[−0.000118, 0.0000130]
Gender	−0.0750^***^	(<0.001)	[−0.0911, −0.0589]	−0.0668^***^	(<0.001)	[−0.0819, −0.0517]
Additional controls	No			Yes		
*N*	17,242			17,242		

*Average marginal effects (ME) with p-values in parentheses and confidence interval (CI) in square brackets*.

* p < 0.05

** p < 0.01

*** *p < 0.001*.

*Panel A: Average marginal effects of pooled probit and random effects probit estimation. Robust standard errors clustered at the individual level (10,559 individuals). Model 1 in each panel contains the following control variables: Gender (female = 1), age, and a wave indicator. Model 2 contains the following additional control variables: Children under six years in the household (yes = 1), education (no degree, lower secondary degree, intermediate secondary degree, high school degree), household income, unemployment (yes = 1)*.

*Source: Own calculations based on NEPS SUF SC6 9.0.1*.

For the interpretation we again focus on the Random Effects Probit models with control variables. We find a recurring pattern for extraversion and openness to experience, both of which are significantly and positively associated with the training probabilities for non-formal as well as for informal further training. The coefficient for openness to new experiences is smaller in the non-formal further training estimation than in the informal training estimation.

Differentiating between training types also reveals different effects for agreeableness, which is positively related to non-formal further training probabilities, but not to informal further training probabilities.

### Gender Differences in Non-formal Further Training

The overall effect differs by gender, as men are more likely to participate in further training, as is shown in Table 6. However, we observe differential gender effects by the type of further training, as becomes evident in Table 7^14^. Women are more likely than men to participate in non-formal further training, but less likely to participate in informal further training. As this result shows interesting gender differences, we investigate these opposing effects more in-depth. Therefore, we estimate the equations with non-formal further training as dependent variable separately for men and women. Table 8 reveals that for both men and women, the results for openness to new experiences remain robust, but the effects of openness to experience are larger for women than they are for men. Additionally, further training decisions of both men and women slightly increase with extraversion.

**Table 8:** Big Five Personality Dimensions and participation in non–formal training by gender.

	**Males**					
	**Pooled Probit model**			**Random Effects Probit model**		
	**Average ME**	***p*–value**	**95% CI**	**Average ME**	***p*-value**	**95% CI**
Extraversion	0.0147^**^	(0.010)	[0.00358, 0.0258]	0.0145^**^	(0.010)	[0.00351, 0.0255]
Agreeableness	0.0104	(0.053)	[−0.000129, 0.0210]	0.00968	(0.069)	[−0.000772, 0.0201]
Conscientiousness	−0.000571	(0.917)	[−0.0113, 0.0102]	−0.000336	(0.951)	[−0.0110, 0.0103]
Neuroticism	0.00271	(0.634)	[−0.00842, 0.0138]	0.00180	(0.749)	[−0.00923, 0.0128]
Openness	0.0178^**^	(0.002)	[0.00663, 0.0290]	0.0181^**^	(0.001)	[0.00702, 0.0291]
Age	0.0215^***^	(<0.001)	[0.0124, 0.0306]	0.0224^***^	(<0.001)	[0.0134, 0.0314]
Age^2^	−0.000278^***^	(<0.001)	[−0.000375, −0.000181]	−0.000287^***^	(<0.001)	[−0.000384, −0.000191]
Additional controls	Yes			Yes		
*N*	8,532			8,532		
	**Females**					
Extraversion	0.0136^*^	(0.015)	[0.00260, 0.0246]	0.0130^*^	(0.019)	[0.00214, 0.0239]
Agreeableness	0.0168^**^	(0.003)	[0.00586, 0.0276]	0.0167^**^	(0.002)	[0.00600, 0.0275]
Conscientiousness	−0.0131^*^	(0.021)	[−0.0243, −0.00202]	−0.0121^*^	(0.030)	[−0.0231, −0.00117]
Neuroticism	−0.0124^*^	(0.024)	[−0.0232, −0.00161]	−0.0137^*^	(0.012)	[−0.0243, −0.00304]
Openness	0.0445^***^	(<0.001)	[0.0338, 0.0553]	0.0430^***^	(<0.001)	[0.0324, 0.0536]
Age	0.0181^***^	(<0.001)	[0.00841, 0.0278]	0.0190^***^	(<0.001)	[0.00935, 0.0287]
Age^2^	−0.000207^***^	(<0.001)	[−0.000310, −0.000103]	−0.000217^***^	(<0.001)	[−0.000320, −0.000114]
Additional controls	Yes			Yes		
*N*	8,710			8,710		

*Average marginal effects (ME) with p-values in parentheses and confidence interval (CI) in square brackets*.

* p < 0.05

** p < 0.01

*** *p < 0.001*.

*Robust standard errors clustered at the individual level. 5,234 males and 5,325 females*.

*All models contain the following additional control variables: Children under six years in the household (yes = 1), education (no degree, lower secondary degree, intermediate secondary degree, high school degree), household income, unemployment (yes = 1) and a wave indicator. The dependent variable is non-formal further training participation (= 1). A suest-test confirms that the genders significantly differ from each other*.

*Source: Own calculations based on NEPS SUF SC6 9.0.1*.

Moreover, we observe gender differences for agreeableness, conscientiousness and neuroticism. Agreeableness positively relates to non-formal further training participation for women only. In contrast, conscientiousness and neuroticism negatively relate to womens', but not mens', non-formal training participation. The marginal effect sizes relate to those of extraversion.

### Privately Motivated Non-formal Further Training

For the subsample of non-formal further training, information on the reasons for partaking in the training activity is available. These reasons can be private or occupationally motivated. Table 9 shows that consistent with Table 1, women are more likely to participate in private further training activities than men. We additionally observe that the direction of the age coefficients reverses.

**Table 9:** Big Five personality dimensions and privately motivated non-formal further training participation.

	**Pooled Probit model**					
	**Model 1**			**Model 2**		
	**Average ME**	***p*-value**	**95% CI**	**Average ME**	***p*-value**	**95% CI**
Extraversion	0.0117	(0.056)	[−0.000295, 0.0238]	0.0115	(0.061)	[−0.000530, 0.0236]
Agreeableness	−0.00289	(0.630)	[−0.0147, 0.00888]	−0.00298	(0.620)	[−0.0148, 0.00881]
Conscientiousness	−0.0199^**^	(0.001)	[−0.0316, −0.00823]	−0.0209^***^	(<0.001)	[−0.0326, −0.00919]
Neuroticism	0.0135^*^	(0.036)	[0.000884, 0.0261]	0.0129^*^	(0.045)	[0.000271, 0.0256]
Openness	0.0212^***^	(<0.001)	[0.00937, 0.0330]	0.0210^***^	(0.001)	[0.00905, 0.0329]
Age	−0.0351^***^	(<0.001)	[−0.0451, −0.0252]	−0.0326^***^	(<0.001)	[−0.0429, −0.0224]
Age^2^	0.000402^***^	(<0.001)	[0.000293, 0.000511]	0.000377^***^	(<0.001)	[0.000265, 0.000488]
Gender	0.0755^***^	(<0.001)	[0.0516, 0.0994]	0.0755^***^	(<0.001)	[0.0514, 0.0997]
Additional controls	No			Yes		
*N*	6,364			6,364		
	**Random Effects Probit Model**					
Extraversion	0.0109	(0.074)	[−0.00107, 0.0229]	0.0109	(0.076)	[−0.00114, 0.0229]
Agreeableness	−0.00152	(0.799)	[−0.0132, 0.0102]	−0.00173	(0.772)	[−0.0134, 0.00998]
Conscientiousness	−0.0191^**^	(0.001)	[−0.0307, −0.00756]	−0.0200^***^	(0.001)	[−0.0316, −0.00837]
Neuroticism	0.0139^*^	(0.028)	[0.00150, 0.0264]	0.0134^*^	(0.034)	[0.000984, 0.0259]
Openness	0.0202^***^	(0.001)	[0.00845, 0.0319]	0.0199^***^	(0.001)	[0.00809, 0.0318]
Age	−0.0350^***^	(<0.001)	[−0.0449, −0.0251]	−0.0325^***^	(<0.001)	[−0.0427, −0.0223]
Age^2^	0.000402^***^	(<0.001)	[0.000293, 0.000510]	0.000376^***^	(<0.001)	[0.000265, 0.000487]
Gender	0.0763^***^	(<0.001)	[0.0525, 0.100]	0.0765^***^	(<0.001)	[0.0525, 0.101]
Additional controls	No			Yes		
*N*	6,364			6,364		

*Average marginal effects (ME) with p-values in parentheses and confidence interval (CI) in square brackets*.

* p < 0.05

** p < 0.01

*** *p < 0.001*.

*Robust standard errors clustered at the individual level (5,067 individuals). Model 1 contains the following control variables: Gender (female = 1), age, and a wave indicator. Model 2 contains the following additional control variables: Children under six years in the household (yes = 1), education (no degree, lower secondary degree, intermediate secondary degree, high school degree), household income, unemployment (yes = 1)*.

*Random sample of respondents with participation in non-formal further training, who were asked whether their non-formal further training was privately motivated (= 1), occupationally motivated (= 0) or both (= 0)*.

*Source: Own calculations based on NEPS SUF SC6 9.0.1*.

Consistent with the previous results, openness to experience positively relates to privately motivated training—albeit with a smaller magnitude. Surprisingly, extraversion does not seem to be associated with participation in privately motivated further training. However, in contrast to Table 8, we now observe that training activities are slightly yet positively associated with neuroticism. Furthermore, conscientiousness negatively relates to privately motivated further training.

### Robustness Checks With Time-Varying Personality Traits

In response to the potential caveat that the means of the individual Big Five Personality Dimensions within individuals may not adequately capture the variability of personality traits, we re-estimate our main regressions with time-varying personality traits. To allow for a detailed comparison between the two methods, the results are displayed in Tables A1–A4. We show that for each regression, the patterns of the Big Five remain the same. Hence, we do not find substantial differences in the estimation results when we estimate the regressions with averages of the Big Five or with time-variant Big Five.

## Discussion

Our analyses based on the Adult Stage of the NEPS reveal a number of findings that expand the existing literature on the relationship between the Big Five personality traits and further training participation.

We exploit the panel character of the dataset and take advantage of yearly measurements of the same individuals, both by averaging repeated measurements to reduce bias from measurement error and by accounting for unobservable heterogeneity by using panel estimators. We show that the relationship between personality and further training participation is not simply a spurious correlation.

We exploit the high-quality data stemming from detailed NEPS questions on different types of further training, as well as its distinction between different reasons for investing in continuous training. Our in-depth-analyses show that differentiating between different training types (i.e., non-formal and informal, as well as work-related and private training) is important, as the five personality traits relate to these training outcomes differently.

We also shed light on gender and age effects for further training participation and highlight that the results are not generalizable over all training types, and hence differentiation is necessary. Finally, we reveal that consistent patterns for personality traits exist across all estimations, namely that openness to new experiences and extraversion positively relate to further training participation, no matter the training type. We discuss these results in detail in this section.

### Age and Further Training

The overlying pattern that emerges from our data with respect to age is that the likelihood to participate in further training increases until middle adulthood—with a peak at nearly 40 years—and then decreases with each additional year. According to human capital theory, older individuals arrive at different cost-benefit calculations because, due to their shorter remaining lifetime and professional career, the returns to educational investments are less likely to exceed their costs. However, when focusing on private training, we find that the sign of the coefficients reverses for private training. This finding indicates that occupational training investments drive the age effect and that the cost-benefit calculations in a private setting are different from those in an occupational context^15^.

Lower costs may also explain this age effect, as individuals grow older and hence may have more time for leisure training activities due to fewer family obligations. This age effect may also indicate that older individuals exploit private further training opportunities to remain up-to-date in terms of social participation. Thus, it seems that societal and private benefits are more likely to outweigh costs with age.

In addition, we explore how the importance of personality traits changes across age. Thereby we calculate the marginal effects of the Random Effects Probit specification (Table 6) for the two significant personality traits—openness to experience and extraversion—at each age. The results as shown in Supplementary Figures 1, 2 illustrate that the marginal effects decrease with age. However, as the confidence bands overlap for each age, this result merely shows a tendency. We presume that the marginal effects are not statistically different from each other, as more observations are needed to conclusively regard the importance of personality across age.

### Personality Traits and Further Training Participation

We first look at overall further training participation i.e., we do not differentiate between different training types in a first step. The results show a positive relationship between extraversion and further training participation, indicating that outgoing and social individuals are more likely to partake in further training than reserved individuals are. Openness to new experiences also positively relates to overall further training participation.

Estimating Pooled Probit models allows us to compare our results with prior results presented by Offerhaus (2012). While we corroborate these earlier results for the positive effect of openness to experience, extraversion was not significant in the previous study.

Compared to the other personality traits, the average marginal effects for openness to experience are larger in magnitude. Thus, openness to experience seems to be the trait most affecting lifelong learning participation decisions. We want to highlight that the marginal effect for openness to experience is smaller in the sample for non-formal training, than it is for overall and informal training. This finding may be driven by the fact that most non-formal further training is occupation-related, as shown in Table 1, where only 27% of the randomly drawn non-formal training are privately motivated. Training activities for occupational reasons may hinge less strongly on openness to experience because the decision to partake in a further training measure is likely not only taken by the employee, but by the employer or at least in accordance with the employer.

When we differentiate between non-formal and informal further training, the main patterns for extraversion and openness to experience remain the same. We also observe a positive relationship between agreeableness and non-formal further training, while this personality trait does not relate to informal further training. We assume that agreeable individuals do not refuse to partake in non-formal courses, particularly as employers often require them. They might however be more reluctant to ask for informal training opportunities.

Overall, we can confirm the importance of openness to experiences for further training participation (Offerhaus 2012). Using recent survey data from the NEPS on adults living in Germany, we show that despite rapid changes in labor market conditions and societal dynamics shaped by digitalization, demographic changes and a post-recession period, the relationship between personality traits and further training holds.

When we look at privately motivated training, we find that openness to experience consistently positively relates to further training participation. Extraversion, however, does not. We additionally find that participation increases with higher scores of neuroticism. This result emphasizes the role of structured training offers, as neurotic individuals may appreciate organized further training in private life to feel more assured and less stressed about their privately motivated endeavors. Furthermore, conscientiousness negatively relates to privately motivated training. We hypothesize that conscientious individuals may not partake in a privately motivated training, when they simultaneously have to meet work requirements. Thus, when job responsibilities and deadlines conflict with a training opportunity, conscientious individuals may favor job requirements over the training.

### Gender Differences in the Relationship Between the Big Five and Further Training

In most specifications, we find that women are less likely to participate in further training. This result is consistent with findings for Switzerland, where women participate less in employer-provided training compared to men. Surprisingly, this finding cannot be explained by part-time work and part-time vs. full-time inequalities (Backes-Gellner et al., 2014). However, in prior results for Germany, summarized by Dietz and Zwick (2020), female training participation seems to be similar to that of men and it is assumed that men are more likely to participate in employer-initiated training, while women seem to be responsible themselves for their training endeavors.

However, we do observe different gender effects when we differentiate between non-formal and informal further training participation. The results from Table 7 indicate that the overall negative effect for women presented in Table 6 is driven by informal further training participation^16^. We propose three possible explanations for these gender differences: First, we suggest that due to working part-time and family obligations, women on average have fewer opportunities to participate in informal training activities both at work and during leisure time. Second, the effect on non-formal training may partly be driven by private training, which women are more likely to attend even during leisure time (compare Table 1). Thus, women who participate in structured classes for leisure activities are likely to drive this result. Third, many regulated occupations, such as for example occupations in the medical sector, require obligatory further training in regular intervals. Given the higher share of women in these occupations, for example in nursing, the obligatory character of further training may drive the results.

Notably, we also find differences for the relationship between the Big Five personality traits and non-formal further training as outcome when we estimate the specification separately for men and women. For women, agreeableness is negatively correlated to non-formal further training participation. Similarly, the marginal effect for conscientiousness is negative. A possible explanation is that highly conscientious women are inclined to prioritize their job or family duties at the expense of training investments. Finally, we also observe a negative relationship between neuroticism and non-formal further training for women. Overall, these results imply that personality traits play a different role for men and women. Particularly it seems that personality traits are more important for women's further training participation than for men's.

### Limitations and Outlook

Our analyses face some limitations, which should be mentioned: First, we do not claim causality with our study, as we only show correlations. Second, while NEPS is of high data quality and allows in-depth investigations of further training participation, the sample is selective in terms of an education bias, which means that we likely regard a sample that is more educated than the population.

Third, we are limited in the measurement of the Big Five. On the one hand, the personality traits were only measured in two waves, which means we might be dealing with measurement error. On the other hand, the Big Five are measured via the short-scale following Rammstedt and John (2007). While the short-scale does not capture as much detailed information as the full scale, it nevertheless has some non-negligible advantages, as it reduces respondent burden and saves time. In addition, previous studies have demonstrated that the short scale captures 70% of the long-scales variance (Rammstedt and John, 2007) and show that short scales are reliable and valid proxies for longer scales (Gosling et al., 2003; Rammstedt and John, 2007). Nevertheless, the short scale lends itself to higher measurement error compared to the long-scale. In addition, due to decreased initial variations and measurement of only two items per personality trait, we may only be estimating lower bounds (Spengler et al., 2013) of the relationship between personality traits and further training participation.

Notably, we find that our main results do not change when we estimate the regressions using wave-specific personality measures instead of the averages of the Big Five. This result may imply that we indeed capture a stable part of personality, which does not change across the waves in our sample. Averages therefore capture the effects of the Big Five well. At the same time, the time lapse between the measurements of the Big Five is not very long (i.e., 3 years) and therefore it might still be possible that personality changes can occur in this sample when a longer period becomes available.

These limitations also imply space for future research, for which we want to highlight some possibilities. While we find that openness to experience is the most important personality trait affecting lifelong learning, we want to stress that other personality traits also matter. Further research is needed to identify the skills most relevant for specific training activities, particularly when regarding training contents and lengths (Janssen and Wölfel, 2017), as these training characteristics may interact with personality. In doing so, the relationship between socio-emotional and cognitive skills should also be taken into account, as non-cognitive and cognitive skills may co-shape competencies (Rammstedt et al., 2017; Lechner et al., 2019c) and thereby future training outcomes. This notion implies that focusing on one personality trait in isolation, such as openness to experience, without enhancing other skills may not yield the desired results.

### Policy Implications

Our results imply two main policy recommendations. First, because we find differential effects for different groups of individuals and personality types, we propose group-specific and even individual-specific further training policies. In addition to obvious groupings along gender and age differences, we highlight the importance of personality differences. Therefore, we suggest personality-specific counseling in addition to differentiations that are more common. For example, adults with low openness to new experiences may need more support from employers or employment agencies to realize the benefits of further training investments. Furthermore, incentives given to individuals to foster further training participation could be modeled to individuals with different personality traits.

Second, we suggest policies that aim at fostering personality traits promoting lifelong learning. As socio-emotional skills change and evolve progressively when children grow into adults, investments into these skills are important, in particular since children with well-developed socio-emotional skills also seem to have an advantage in building cognitive skills (OECD, 2019). Thus, based on empirical evidence on the malleability of personality traits in early phases of the lifecycle and the possibility to strengthen traits in childhood, we suggest addressing policies toward individuals early in life to lay the foundation children for lifelong learning^17^.

Overall, a one-size fits-all approach may not work and more differentiated policy approaches are needed to foster both favorable socio-emotional skills early on and continuing learning over the whole life course.

## Conclusion

We investigate the relationship between personality traits and further training participation for occupational *and* private reasons and for different training types, namely non-formal and informal training measures. Based on data from the NEPS, we show that the Big Five Personality Dimensions play an important role for the further training participation decision of adults. Irrespective of the type of further training and of the motivation for the training, openness to new experiences and extraversion show a strong positive relationship with further training probabilities. The importance of the remaining four personality traits differ with the type of further training chosen, and with the motive behind further training (i.e., occupational vs. private training activities). Additionally, gender differences in the magnitude and significance become apparent for different personality traits, particularly for non-formal and informal further training. Despite the heterogeneous effects of the individual Big Five Personality Dimensions, we conclude that personality is an important determinant of further training activities.

We contribute to the literature by exploiting the high quality panel data of the NEPS Adult Cohort, which allows us conducting in-depth-analyses and controlling for unobserved heterogeneity. Thereby, we present first results showing that the relationship between personality and further training is not simply a spurious correlation. Our findings indicate that the distinction between further training activities is important to understand which personality traits are associated with different training decisions. In the context of the labor market, our results indicate that occupational further training is a possible channel to explain the importance of personality traits, in particular openness to experience, for labor market success. Personality also plays a role in lifelong learning in a private setting and has the potential to improve life outcomes, leisure activities and societal participation.

## Data Availability Statement

This paper uses data from the National Educational Panel Study (NEPS): Starting Cohort Adults, 10.5157/NEPS:SC6:9.0.1. From 2008 to 2013, NEPS data was collected as part of the Framework Program for the Promotion of Empirical Educational Research funded by the German Federal Ministry of Education and Research (BMBF). As of 2014, NEPS is carried out by the Leibniz Institute for Educational Trajectories (LIfBi) at the University of Bamberg in cooperation with a nationwide network. All documentation concerning NEPS and including questionnaires and data manuals are made available by the LIfBi (https://www.neps-data.de/Data-Center/Data-and-Documentation/Starting-Cohort-Adults/Documentation). A list of publications with NEPS SC6 data is equally available from the LIfBi (https://www.neps-data.de/Project-Overview/Publications). The NEPS data is available to the research community via the Research Data Center of the LIfBi (https://www.neps-data.de/Data-Center/Data-Access). Due to the German Data Protection legislation, we cannot make the original NEPS data or the dataset we generated available. Researchers can however apply for data access via the LIfBi. We will make all our do-files available to researchers upon request for replication studies.

## Ethics Statement

The NEPS study is conducted under the supervision of the German Federal Commissioner for Data Protection and Freedom of Information (BfDI) and in coordination with the German Standing Conference of the Ministers of Education and Cultural Affairs (KMK) and - in the case of surveys at schools - the Educational Ministries of the respective Federal States. All data collection procedures, instruments, and documents were checked by the data protection unit of the Leibniz Institute for Educational Trajectories (LIfBi). The necessary steps are taken to protect participants' confidentiality according to national and international regulations of data security. Participation in the NEPS study is voluntary and based on the informed consent of participants. This consent to participate in the NEPS study can be revoked at any time.

## Author Contributions

SA, MB, and M-CL developed the idea, discussed the structure, content of the paper, and interpreted the results together. MB ran the estimations. M-CL wrote the first draft of the paper and the revised version. SA and M-CL refined the manuscript. All authors contributed to the article and approved the submitted version.

## Conflict of Interest

The authors declare that the research was conducted in the absence of any commercial or financial relationships that could be construed as a potential conflict of interest.

## Footnotes

^1^For example, in 2018, the shortage of skilled workers has reached its peak in some industries and regions in Germany (Dettmann et al., 2018). A key competitive advantage for the future of skilled labor lies in developing the skills of the existing workforce not only through initial vocational training, but also through further training.

^2^The standard retirement age in Germany will be increasing to 67 years by 2029, and modifications to the German legislation allow more flexible models of working beyond the standard age for entry into the pension system. More than a quarter of retirees work in the three years after having reached the standard retirement age (Anger et al., 2018).

^3^For an earlier and similar definition of personality traits see (Roberts, 2009, p. 7).

^4^Almlund et al. (2011) provide an earlier and very comprehensive overview.

^5^Preloads refer to cues from previous interviews, such as a start date of an employment spell or the occupation given in the last interview.

^6^The Adult Stage originates in the survey “Working and Learning in a Changing World” (ALWA) run by the Institute for Employment Research (IAB) in 2007/2008 and integrated into NEPS in 2009 (Allmendinger et al., 2019).

^7^Through this restriction, we only exclude 0.54% of individuals from the sample.

^8^The original question allows a third answer option “both privately and occupationally motivated”. We recode this option to be occupationally motivated, as we want to separate out fully privately motivated further training activities.

^9^The difference is significant at the 0.001 significance level.

^10^However, including individuals with information on personality in only one wave virtually produces the same virtually produces the same results. Therefore we use these individuals with only one observation in our regression analyses.

^11^To be precise, we are not able to differentiate between possible age and cohort effects. Any differences in personality between birth cohorts will also be picked up by the age-correction in our sample.

^12^Note that the Big Five will be treated as time-variant, when we rerun the regressions in a robustness check, where we relax the assumption of stability in the traits and use the wave-specific Big Five instead.

^13^Note that we use Random Effects as opposed to Fixed Effects estimators, as the Fixed Effect's identification relies on the time-variation of our variables. As the fixed effects estimate a de-meaned model by subtracting the average of the period for each individual, many of our variables would be eliminated, as they do not vary with time, such as the Big Five, gender and education. The Random Effects model treats unobserved individual effects as stochastic variable, whereas the Fixed Effects model treats it as time constant effects (Wooldridge, 2010).

^14^A suest-test shows that the gender differences are statistically significant (chi squared = 117.89).

^15^In addition to the differential outcomes of privately motivated and work-related training, the costs may vary substantially both in size and in the financial burden to the individual, in particular when private training is compared with employer-provided training measures.

^16^Note, that the gender effect merely reflects the number of trainings. A report shows that men and women additionally differ in training content and length (Janssen and Wölfel, 2017).

^17^For example, Heckman et al. (2013) show that the long-term success of an early childhood intervention program can be primarily attributed to lasting changes in non-cognitive skills. Additional evidence suggests that supporting children and teenagers through mentoring programs (Kosse et al., 2019) promotes personality development. An extensive overview of interventions to foster non-cognitive skills for all possible age groups (Kautz et al., 2014) further suggest that comprehensive interventions need to consider contextual factors, the desired outcomes and the outcome-determining personality traits in order to successfully prepare individuals for the future.
