# Supplementary material for: Personality Traits and Further Training
Source: Front Psychol. 2020 Nov 16;11:510537. doi: 10.3389/fpsyg.2020.510537 (PMC7701053; doi:10.3389/fpsyg.2020.510537)
Supplement: Supplementary file 1 [file Data_Sheet_1.pdf]

Personality Traits and Further Training

Marie-Christine Laible, Silke Anger, Martina Baumann

Supplementary Materials: Appendix

Table A1: Big Five Personality Dimensions and Participation in Overall Further Training

Panel A: Pooled OLS (top) and Random Effects OLS (bottom)

	Pooled OLS					
	Model 1			Model 2		
	Average ME	p-value	95%CI	Average ME	p-value	95%CI
Extraversion	0.0123***	(< .001)	[0.00572,0.0190]	0.0139***	(< .001)	[0.00757,0.0203]
Agreeableness	0.00236	(.476)	[-0.00413,0.00886]	0.00488	(.126)	[-0.00137,0.0111]
Conscientiousness	-0.0133***	(< .001)	[-0.0197,-0.00687]	-0.00380	(.227)	[-0.00995,0.00236]
Neuroticism	-0.0102**	(.003)	[-0.0169,-0.00348]	-0.00349	(.287)	[-0.00992,0.00294]
Openness	0.0696***	(< .001)	[0.0632,0.0759]	0.0540***	(< .001)	[0.0479,0.0602]
Age	0.0217***	(< .001)	[0.0165,0.0270]	0.00871**	(.001)	[0.00348,0.0139]
Age^2	-0.000277***	(< .001)	[-0.000334,-0.000220]	-0.000123***	(< .001)	[-0.000180,-0.0000666]
Gender	-0.0509***	(< .001)	[-0.0639,-0.0379]	-0.0433***	(< .001)	[-0.0558,-0.0308]
Additional controls	no			yes		
N	17,242			17,242		
	Random Effects OLS					
Extraversion	0.0120***	(.001)	[0.00507,0.0190]	0.0137***	(< .001)	[0.00708,0.0204]
Agreeableness	0.00357	(.302)	[-0.00321,0.0104]	0.00552	(.098)	[-0.00102,0.0121]
Conscientiousness	-0.00989**	(.004)	[-0.0165,-0.00325]	-0.00212	(.515)	[-0.00850,0.00426]
Neuroticism	-0.00941**	(.008)	[-0.0163,-0.00249]	-0.00382	(.260)	[-0.0105,0.00283]
Openness	0.0622***	(< .001)	[0.0554,0.0690]	0.0492***	(< .001)	[0.0427,0.0557]
Age	0.0209***	(< .001)	[0.0153,0.0266]	0.00913**	(0.001)	[0.00356,0.0147]
Age^2	-0.000269***	(< .001)	[-0.000330,-0.000208]	-0.000127***	(< .001)	[-0.000187,-0.0000668]
Gender	-0.0511***	(< .001)	[-0.0656,-0.0366]	-0.0438***	(< .001)	[-0.0576,-0.0301]
Additional controls	no			yes		
N	17,242			17,242		

Panel B: Pooled Probit (top) and Random Effects Probit (bottom)

	Pooled Probit					
	Model 1			Model 2		
	Average ME	p-value	95%CI	Average ME	p-value	95%CI
Extraversion	0.0123***	(.001)	[0.00535,0.0192]	0.0132***	(< .001)	[0.00675,0.0197]
Agreeableness	0.00323	(.352)	[-0.00358,0.0100]	0.00539	(.096)	[-0.000966,0.0117]
Conscientiousness	-0.0134***	(< .001)	[-0.0202,-0.00650]	-0.00361	(.268)	[-0.00999,0.00277]
Neuroticism	-0.0100**	(.005)	[-0.0170,-0.00309]	-0.00312	(.343)	[-0.00955,0.00332]
Openness	0.0682***	(< .001)	[0.0616,0.0747]	0.0518***	(< .001)	[0.0456,0.0580]
Age	0.0199***	(< .001)	[0.0143,0.0254]	0.00801**	(.005)	[0.00247,0.0136]
Age^2	-0.000255***	(< .001)	[-0.000314,-0.000195]	-0.000112***	(< .001)	[-0.000171,-0.0000532]
Gender	-0.0499***	(< .001)	[-0.0643,-0.0355]	-0.0420***	(< .001)	[-0.0556,-0.0285]
Additional controls	no			yes		
N	17,242			17,242		
	**Random Effects Probit**					
Extraversion	0.0122***	(< .001)	[0.00540,0.0190]	0.0131***	(< .001)	[0.00674,0.0195]
Agreeableness	0.00431	(.202)	[-0.00231,0.0109]	0.00599	(.059)	[-0.000225,0.0122]
Conscientiousness	-0.0106**	(.002)	[-0.0173,-0.00391]	-0.00246	(.442)	[-0.00874,0.00381]
Neuroticism	-0.00925**	(.007)	[-0.0160,-0.00254]	-0.00339	(.289)	[-0.00966,0.00288]
Openness	0.0616***	(< .001)	[0.0551,0.0681]	0.0476***		[0.0414,0.0538]
Age	0.0197***	(< .001)	[0.0141,0.0252]	0.00857**	(.002)	[0.00308,0.0141]
Age^2	-0.000252***	(< .001)	[-0.000311,-0.000193]	-0.000117***	(< .001)	[-0.000176,-0.0000592]
Gender	-0.0500***	(< .001)	[-0.0644,-0.0356]	-0.0419***	(< .001)	[-0.0554,-0.0284]
Additional controls	no			yes		
N	17,242			17,242		

Notes: Average marginal effects (ME) with p-values in parentheses and confidence interval (CI) in square brackets. * $p < .05$, ** $p < .01$, *** $p < .001$. Panel A: Average marginal effects of pooled ordinary least squares (OLS) and random effects estimation. Robust standard errors clustered at the individual level (10,559 individuals). Panel B: Average marginal effects of pooled probit and random effects probit estimation. Robust standard errors clustered at the individual level (10,559 individuals). Model 1 in each panel contains the following control variables: Gender (female=1), age, and a wave indicator. Model 2 contains the following additional control variables: Children under six years in the household (yes =1), education (no degree, lower secondary degree, intermediate secondary degree, high school degree), household income, unemployment (yes=1).
Source: Own calculations based on NEPS SUF SC6 9.0.1.

Table A2: Big Five Personality Dimensions and Non-Formal and Informal Further Training Participation

Panel A: Participation in Non-Formal Training

	**Pooled Probit**					
	Model 1			Model 2		
	Average ME	p-value	95%CI	Average ME	p-value	95%CI
Extraversion	0.0141***	(< .001)	[0.00621,0.0221]	0.0143***	(0.000)	[0.00652,0.0222]
Agreeableness	0.0109**	(0.005)	[0.00327,0.0186]	0.0132***	(0.001)	[0.00559,0.0208]
Conscientiousness	-0.0123**	(0.002)	[-0.0201,-0.00445]	-0.00710	(0.073)	[-0.0148,0.000653]
Neuroticism	-0.0103*	(0.010)	[-0.0181,-0.00243]	-0.00547	(0.167)	[-0.0132,0.00229]
Openness	0.0402***	(< .001)	[0.0324,0.0480]	0.0320***	(0.000)	[0.0243,0.0398]
Age	0.0318***	(< .001)	[0.0253,0.0383]	0.0205***	(< .001)	[0.0139,0.0271]
Age^2	-0.000380***	(< .001)	[-0.000449,-0.000310]	-0.000250***	(< .001)	[-0.000321,-0.000179]
Gender	0.0449***	(< .001)	[0.0285,0.0614]	0.0521***	(< .001)	[0.0359,0.0683]
Additional controls	no			yes		
N	17,242			17,242		

	**Random Effects Probit**					
Extraversion	0.0135***	(.001)	[0.00565,0.0213]	0.0139***	(< .001)	[0.00620,0.0217]
Agreeableness	0.0105**	(.006)	[0.00297,0.0181]	0.0127***	(.001)	[0.00525,0.0202]
Conscientiousness	-0.0112**	(.004)	[-0.0189,-0.00350]	-0.00650	(.096)	[-0.0142,0.00116]
Neuroticism	-0.0110**	(.005)	[-0.0187,-0.00329]	-0.00651	(.096)	[-0.0142,0.00116]
Openness	0.0393***	(< .001)	[0.0316,0.0469]	0.0314***	(< .001)	[0.0237,0.0391]
Age	0.0322***	(< .001)	[0.0258,0.0386]	0.0212***	(< .001)	[0.0146,0.0278]
Age^2	-0.000385***	(< .001)	[-0.000453,-0.000316]	-0.000259***	(< .001)	[-0.000329,-0.000188]
Gender	0.0449***	(< .001)	[0.0286,0.0613]	0.0518***	(< .001)	[0.0357,0.0679]
Additional controls	no			yes		
N	17,242			17,242		

Panel B: Participation in Informal Training

	Pooled Probit					
	Model 1			Model 2		
	Average ME	p-value	95%CI	Average ME	p-value	95%CI
Extraversion	0.0101*	(.011)	[0.00236,0.0179]	0.0125***	(.001)	[0.00524,0.0199]
Agreeableness	0.00276	(.475)	[-0.00482,0.0103]	0.00505	(.162)	[-0.00203,0.0121]
Conscientiousness	-0.0110**	(.005)	[-0.0186,-0.00338]	0.00114	(.753)	[-0.00598,0.00826]
Neuroticism	-0.0100*	(.011)	[-0.0178,-0.00231]	-0.00250	(.496)	[-0.00970,0.00470]
Openness	0.0793***	(< .001)	[0.0721,0.0866]	0.0596***	(< .001)	[0.0526,0.0666]
Age	0.0145***	(< .001)	[0.00808,0.0208]	0.00323	(.313)	[-0.00304,0.00951]
Age^2	-0.000190***	(< .001)	[-0.000258,-0.000122]	-0.0000509	(.135)	[-0.000118,0.0000158]
Gender	-0.0742***	(< .001)	[-0.0904,-0.0581]	-0.0664***	(< .001)	[-0.0816,-0.0512]
Additional controls	no			yes		
N	17,242	17,242				

	Random Effects Probit					
Extraversion	0.0108**	(.005)	[0.00323,0.0183]	0.0128***	(< .001)	[0.00572,0.0200]
Agreeableness	0.00439	(.240)	[-0.00293,0.0117]	0.00610	(.083)	[-0.000796,0.0130]
Conscientiousness	-0.00831*	(.026)	[-0.0157,-0.000973]	0.00178	(.614)	[-0.00515,0.00872]
Neuroticism	-0.00788*	(.036)	[-0.0153,-0.000495]	-0.00196	(.580)	[-0.00892,0.00500]
Openness	0.0686***	(< .001)	[0.0614,0.0759]	0.0526***	(< .001)	[0.0456,0.0595]
Age	0.0137***	(< .001)	[0.00737,0.0199]	0.00344	(0.275)	[-0.00274,0.00962]
Age^2	-0.000182***	(< .001)	[-0.000249,-0.000115]	-0.0000526	(0.116)	[-0.000118,0.0000130]
Gender	-0.0750***	(< .001)	[-0.0911,-0.0589]	-0.0668***	(< .001)	[-0.0819,-0.0517]
Additional controls	no			yes		
N	17,242			17,242		

Notes: Average marginal effects (ME) with p-values in parentheses and confidence interval (CI) in square brackets. * $p < .05$, ** $p < .01$, *** $p < .001$. Panel A: Average marginal effects of pooled probit and random effects probit estimation. Robust standard errors clustered at the individual level (10,559 individuals). Model 1 in each panel contains the following control variables: Gender (female=1), age, and a wave indicator. Model 2 contains the following additional control variables: Children under six years in the household (yes =1), education (no degree, lower secondary degree, intermediate secondary degree, high school degree), household income, unemployment (yes=1).
Source: Own calculations based on NEPS SUF SC6 9.0.1.

Table A3: Big Five Personality Dimensions and Participation in Non-Formal Training by Gender

	**Male**					
	Model 1			Model 2		
	Average ME	p-value	95%CI	Average ME	p-value	95%CI
Extraversion	0.0147**	(.010)	[0.00358,0.0258]	0.0145**	(.010)	[0.00351,0.0255]
Agreeableness	0.0104	(.053)	[-0.000129,0.0210]	0.00968	(.069)	[-0.000772,0.0201]
Conscientiousness	-0.000571	(.917)	[-0.0113,0.0102]	-0.000336	(.951)	[-0.0110,0.0103]
Neuroticism	0.00271	(.634)	[-0.00842,0.0138]	0.00180	(.749)	[-0.00923,0.0128]
Openness	0.0178**	(.002)	[0.00663,0.0290]	0.0181**	(.001)	[0.00702,0.0291]
Age	0.0215***	(< .001)	[0.0124,0.0306]	0.0224***	(< .001)	[0.0134,0.0314]
Age^2	-0.000278***	(< .001)	[-0.000375,-0.000181]	-0.000287***	(< .001)	[-0.000384,-0.000191]
Additional controls	yes			yes		
N	8,532			8,532		

	**Female**					
Extraversion	0.0136*	(.015)	[0.00260,0.0246]	0.0130*	(.019)	[0.00214,0.0239]
Agreeableness	0.0168**	(.003)	[0.00586,0.0276]	0.0167**	(.002)	[0.00600,0.0275]
Conscientiousness	-0.0131*	(.021)	[-0.0243,-0.00202]	-0.0121*	(.030)	[-0.0231,-0.00117]
Neuroticism	-0.0124*	(.024)	[-0.0232,-0.00161]	-0.0137*	(.012)	[-0.0243,-0.00304]
Openness	0.0445***	(< .001)	[0.0338,0.0553]	0.0430***	(< .001)	[0.0324,0.0536]
Age	0.0181***	(< .001)	[0.00841,0.0278]	0.0190***	(< .001)	[0.00935,0.0287]
Age^2	-0.000207***	(< .001)	[-0.000310,-0.000103]	-0.000217***	(< .001)	[-0.000320,-0.000114]
Additional controls	yes			yes		
N	8,710			8,710		

Notes: Average marginal effects (ME) with p-values in parentheses and confidence interval (CI) in square brackets. * p < .05, ** p < .01, *** p < .001. Robust standard errors clustered at the individual level: 5,234 males and 5,325 females. All models contain the following additional control variables: Children under six years in the household (yes =1), education (no degree, lower secondary degree, intermediate secondary degree, high school degree), household income, unemployment (yes=1) and a wave indicator. The dependent variable is non-formal further training participation (=1). A suest-test confirms that the genders significantly differ from each other.
Source: Own calculations based on NEPS SUF SC6 9.0.1.

Table A4 Big Five Personality Dimensions and Privately Motivated Non-Formal Further Training Participation

	Pooled Probit					
	Model 1			Model 2		
	Average ME	p-value	95%CI	Average ME	p-value	95%CI
Extraversion	0.0117	(.056)	[-0.000295,0.0238]	0.0115	(.061)	[-0.000530,0.0236]
Agreeableness	-0.00289	(.630)	[-0.0147,0.00888]	-0.00298	(.620)	[-0.0148,0.00881]
Conscientiousness	-0.0199***	(.001)	[-0.0316,-0.00823]	-0.0209***	(< .001)	[-0.0326,-0.00919]
Neuroticism	0.0135*	(.036)	[0.000884,0.0261]	0.0129*	(.045)	[0.000271,0.0256]
Openness	0.0212***	(< .001)	[0.00937,0.0330]	0.0210***	(.001)	[0.00905,0.0329]
Age	-0.0351***	(< .001)	[-0.0451,-0.0252]	-0.0326***	(< .001)	[-0.0429,-0.0224]
Age^2	0.000402***	(< .001)	[0.000293,0.000511]	0.000377***	(< .001)	[0.000265,0.000488]
Gender	0.0755***	(< .001)	[0.0516,0.0994]	0.0755***	(< .001)	[0.0514,0.0997]
Additional controls	no			yes		
N	6,364			6,364		
	**Random Effects Probit**					
Extraversion	0.0109	(.074)	[-0.00107,0.0229]	0.0109	(.076)	[-0.00114,0.0229]
Agreeableness	-0.00152	(.799)	[-0.0132,0.0102]	-0.00173	(.772)	[-0.0134,0.00998]
Conscientiousness	-0.0191**	(.001)	[-0.0307,-0.00756]	-0.0200***	(.001)	[-0.0316,-0.00837]
Neuroticism	0.0139*	(.028)	[0.00150,0.0264]	0.0134*	(.034)	[0.000984,0.0259]
Openness	0.0202***	(.001)	[0.00845,0.0319]	0.0199***	(.001)	[0.00809,0.0318]
Age	-0.0350***	(< .001)	[-0.0449,-0.0251]	-0.0325***	(< .001)	[-0.0427,-0.0223]
Age^2	0.000402***	(< .001)	[0.000293,0.000510]	0.000376***	(< .001)	[0.000265,0.000487]
Gender	0.0763***	(< .001)	[0.0525,0.100]	0.0765***	(< .001)	[0.0525,0.101]
Additional controls	no			yes		
N	6,364			6,364		

Notes: Average marginal effects (ME) with p-values in parentheses and confidence interval (CI) in square brackets. * $p < .05$, ** $p < .01$, *** $p < .001$. Robust standard errors clustered at the individual level (5,067 individuals). Model 1 contains the following control variables: Gender (female=1), age, and a wave indicator. Model 2 contains the following additional control variables Children under six years in the household (yes =1), education (no degree, lower secondary degree, intermediate secondary degree, high school degree), household income, unemployment (yes=1). Random sample of respondents with participation in non-formal further training, who were asked whether their non-formal further training was privately motivated (=1), occupationally motivated (=0) or both (=0).

Source: Own calculations based on NEPS SUF SC6 9.0.1

Figure 1: Extraversion and Further Training Participation Across Age

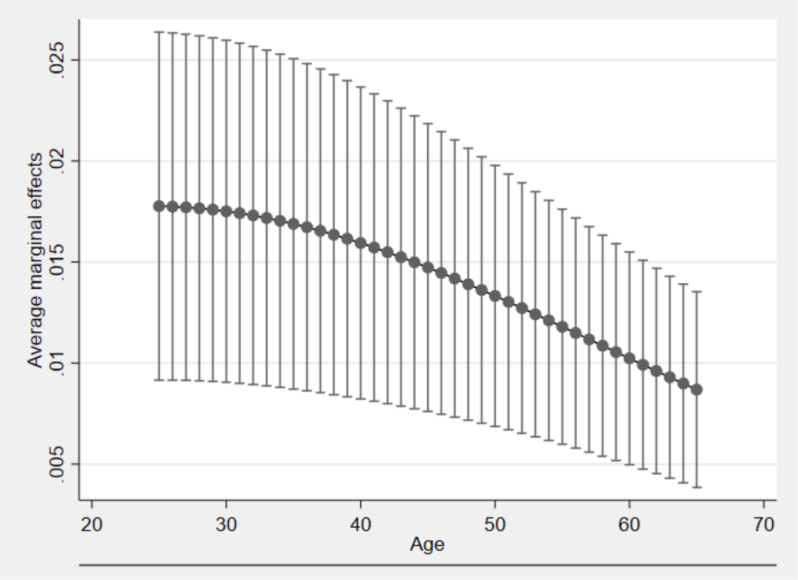

Notes: This figure shows the average marginal effects and the 95% confidence intervals of the impact of extraversion on further training participation across age for N=17,242 individuals.
Source: Own calculations based on NEPS SUF SC6 9.0.1.

Figure 2: Openness and Further Training Participation Across Age

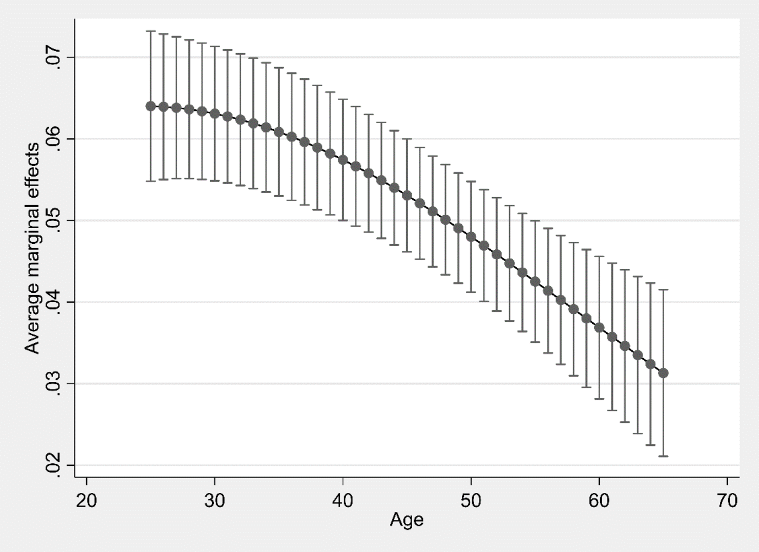

Notes: This figure shows the average marginal effects and the 95% confidence intervals of the impact of openness on further training participation across age for N=17,242 individuals.
